# Neuromodulation of Foraging Decisions: The Role of Dopamine

**DOI:** 10.3389/fnbeh.2021.660667

**Published:** 2021-04-13

**Authors:** Anna Marzecová, Luca F. Kaiser, Armin Maddah

**Affiliations:** ^1^Department of Experimental Psychology, Ghent University, Ghent, Belgium; ^2^Institute of Experimental Psychology, Heinrich-Heine University, Düsseldorf, Germany

**Keywords:** decision making, dopamine, foraging, neuromodulation, patch-leaving

When searching for food, animals need to decide whether they can maximize rewards by harvesting at a current resource, or whether they should instead leave for another foraging site. Humans face similar types of problems when deciding whether to stay with their current job, or to move to a new one with a prospect of better career opportunities. Such decisions to leave, often referred to as patch-leaving decisions, require dynamically weighing the time and energy costs of leaving, as well as the benefits of encountering more rewarding resources at new locations. How neuromodulators are involved in patch-leaving decisions, especially in humans, is, at present, scarcely researched. In their recent study, Le Heron et al. (2020) fill this gap by investigating how these decisions are causally affected by dopaminergic state in an ecologically valid foraging scenario. In their study, participants could choose between collecting reward (milk filling a bucket) at one location (patch) or leaving for another patch which incurred a cost in the form of a fixed travel time. As soon as participants started harvesting (collecting milk) from one patch, the reward per time in that patch decreased exponentially, emulating a depleting resource. To maximize their reward rate, participants were thus faced with the task of continuously comparing the rewards at current location against potential rewards at other locations, whilst taking into account the time cost for leaving.

The optimum solution to this foraging problem is given by the Marginal Value Theorem (MVT, Charnov, 1976; Stephens and Krebs, 1986), which has been shown to predict foraging behavior in many species (Cassini et al., 1993; Hayden et al., 2011). MVT states that the optimal time to leave the current patch is when its marginal reward rate (“foreground”) drops below the average reward rate in the environment (“background”). To separately manipulate background and foreground reward rates, the authors created patches that differed in their (initial) reward rates (low, medium, and high yield). These could be encountered in either a rich or poor environment. In the rich environment, participants were most likely to transition to a high yield patch upon leaving the current patch, whereas in the poor environment, encountering a low yield patch was most likely. The reward obtained in the current patch thus constituted the foreground, whereas the proportion of the different patch types determined the background reward rate. MVT predicts that optimally behaving agents will.

H1: leave patches within an environment (i.e., equal background) at the same reward rate for all patch types; therefore leave patches with lower initial foreground reward earlier than patches with higher foreground reward.

H2: leave earlier in general when in rich compared to poor environments (high vs. low background reward rate).

A main effect of background reward rate on patch leaving times was observed, supporting H2. In contrast, pertaining to H1, participants left patches with lower foreground reward rate earlier, but they seemed to exhibit a tendency to stay longer in high yield patches, in contrast with the prediction that at leaving, the foreground rate is the same for all patch types. Additionally, participants stayed in patches longer than optimal (“overharvested”) across all patch types, leading to less reward obtained than predicted by MVT. Overharvesting is a phenomenon reported ubiquitously in the foraging literature (see e.g., Hayden et al., 2011; Kane et al., 2019) and has been related to different factors, including time preferences (Kane et al., 2019), and behavioral variability (Cash-Padgett and Hayden, 2020).

Evidence on neuromodulatory mechanisms underlying value comparisons in foraging environments remains scant. Tonic dopamine (DA) levels have been previously suggested to scale with the average background reward rate (average of prediction errors) in the environment (Niv et al., 2007; Beierholm et al., 2013) and could therefore be considered a key element in signaling decisions to leave a patch (Constantino et al., 2017). Le Heron et al. (2020) thus hypothesized that tonic DA levels would modulate the impact of the background, but not the foreground reward rate on patch-leaving decisions. To test this hypothesis, a group of elderly participants was tested twice on the foraging task under the influence of either placebo or the D2 receptor agonist cabergoline. When “on” cabergoline, participants left patches in the poor environment earlier. In contrast, cabergoline did not modulate the effect of the foreground reward rate on patch leaving. This pattern resonates well with the hypothesized role of tonic DA in encoding the average background reward rate. Since participants generally overharvested, this may also imply a shift toward more optimal behavior.

A 1 mg dose of cabergoline was hypothesized to specifically influence the perceived background reward by increasing tonic DA levels, acting via postsynaptic mechanisms (Brooks et al., 1998). However, there have been discussions of whether similar doses of D2 agonists would instead impact phasic rather than tonic DA signaling (Santesso et al., 2009; Norbury et al., 2013) through a modulation of presynaptic autoreceptors (Frank and O'Reilly, 2006). Given that there has been no possibility to assess pre- vs. post-synaptic medication effects in the current study, one may not exclude the possibility that the cabergoline dose resulted in a reduction of the phasic tone (Frank and O'Reilly, 2006). A recent study has shown that a reduction of phasic DA may lead to an increase in (random) exploration (Cinotti et al., 2019), and could thus promote patch-leaving behavior. Contributions of both the phasic and the tonic mode in modulating perceived background reward rate may be considered, bearing in mind it has recently been suggested that the distinction between tonic and phasic DA release and its relation to behavior may not be as clear-cut as previously thought (Berke, 2018).

In another recent study, DA depletion associated with Parkinson's disease (PD), has been linked to a lower estimate of background reward rates in a previous study. PD patients overharvested to a larger extent than control participants when “off” DA medication, while their performance was comparable to controls when “on” medication (Constantino et al., 2017). In that study, the richness of the environment varied due to long and short travel costs. Notably, the difference in leaving time between control and PD participants was more pronounced in the richer (short travel) environment. This may imply multiplicative effects on the perceived richness of the environment, but contrasts with Le Heron et al. (2020) finding of effects in poorer environments only. Since participants in both studies discussed above can be assumed to differ with respect to their baseline DA levels, and potential compensatory changes to DA systems, different ceiling effects may have brought about differing patterns of results. Noteworthy, Le Heron et al. (2020) increased DA levels by targeting D2 receptors, while the depletion of DA in PD is likely to affect both D1 and D2 type receptors (Seeman and Niznik, 1990). However, D2 receptors, owing to their higher affinity for DA, may be still sensitive to (subtle) variations in DA concentration in PD patients. Additionally, whether the effects of DA manipulation extend to younger healthy populations (with likely higher baseline DA levels) is an open question. Future work should seek to delineate under which specific circumstances DA modulates the influence of perceived environmental richness on behavior.

Importantly, the specific drug effects might potentially be considered in relation to different manipulations of environmental richness in the two studies. According to MVT, the background reward rate is determined by the value of potential alternatives as well as by costs of accessing these options. During traveling, the net reward intake is zero, therefore the agent needs to consider whether the potential benefits in alternative patches are worth the invested cost of time (i.e., the foregone reward while traveling). As in Constantino et al. (2017) study, decreasing travel time costs should lead to earlier patch leaving, since it translates into an increased background reward rate. Travel times have been previously found to influence the leaving threshold in patch leaving tasks (Hayden et al., 2011; Wolfe, 2013; Ramakrishnan et al., 2019). In the study by Le Heron et al. (2020), travel costs were kept constant in both environments. However, since the average expected reward rate is different in both environments, the opportunity costs of time differ. The equal travel times therefore potentially have a distinct effect in poor and rich environments. While the relationship between DA modulations and subjective travel cost estimates has been scarcely addressed so far (Constantino et al., 2017), there is a rich literature about the effects of DA on cost-benefit decisions (Salamone et al., 1994; Beeler and Mourra, 2018). In these paradigms, subjects usually decide whether a potential outcome is worth a certain effort, which is a conceptually similar question as in the reported foraging scenario: “Is my investment worth the expected payoff?”. A potential route to an increase in the subjective estimate of environmental richness may be a decrease in the subjective estimate of the opportunity costs of time. It would be interesting for further research to explicitly vary travel time costs to assess the contribution of costs to estimates of environmental richness. Combining the experimental manipulations of travel time costs (Constantino et al., 2017) and patch reward yield proportions determining environmental richness (Le Heron et al., 2020) could thus prove useful to further a comprehensive framework on how DA modulates patch-leaving. To build a full picture of dopaminergic control of patch-leaving behavior, future research should systematically consider pharmacological effects of particular drug manipulations, behavioral consequences of experimental manipulations, and the extent to which learning takes place in the task.

Research on the role of other neuromodulators implicated in patch-leaving decisions has started to emerge. The locus-coeruleus (LC) noradrenaline system may be involved in patch-leaving, as it promotes behavioral flexibility (Aston-Jones and Cohen, 2005). A recent study reported that tonic LC stimulation in rats led to an earlier patch leaving, which was related to an increased decision noise (Kane et al., 2017). Conversely, optogenetic stimulation of serotonergic cells in the dorsal raphe nucleus led to later leaving times in a patch leaving task (Lottem et al., 2018). Additionally, a recent whole-brain imaging evidence showing that persistent serotonergic activity correlates with a state of exploitation (Marques et al., 2020). Furthermore, GABA and glutamate concentrations in the anterior cingulate cortex have been shown to predict patch-leaving behavior in healthy participants (Kaiser et al., 2021).

Understanding patch leaving decisions and their underlying neurochemical mechanisms is of fundamental relevance to understanding many neuropsychiatric disorders (Addicott et al., 2015). Le Heron et al. (2020) and Constantino et al. (2017) therefore provide new evidence of high practical importance by exploring a modulatory role of DA in the encoding of background reward rates in patch leaving decisions.

## Author Contributions

AMar: conceptualization (equal), investigation (equal), writing-original draft (lead), writing-review, and editing (equal). LK: conceptualization (equal), investigation (equal), writing-original draft (supporting), writing-review, and editing (equal). AMad: conceptualization (equal), investigation (supporting), writing-review, and editing (equal). All authors contributed to the article and approved the submitted version.

## Conflict of Interest

The authors declare that the research was conducted in the absence of any commercial or financial relationships that could be construed as a potential conflict of interest.
